# Cathepsin W, T-cell receptor-associated transmembrane adapter 1, lymphotactin and killer cell lectin like receptor K1 are sensitive and specific RNA biomarkers of canine epitheliotropic lymphoma

**DOI:** 10.3389/fvets.2023.1225764

**Published:** 2023-11-03

**Authors:** Jadesola Temitope Olayinka, Akanksha Nagarkar, Diana Junyue Ma, Neil B. Wong, Andrew Romasco, Cesar Piedra-Mora, Linda Wrijil, Clement N. David, Heather L. Gardner, Nicholas A. Robinson, Kelly L. Hughes, Bruce Barton, Cheryl A. London, Ramón M. Almela, Jillian M. Richmond

**Affiliations:** ^1^Department of Dermatology, UMass Chan Medical School, Worcester, MA, United States; ^2^SUNY Downstate School of Medicine, New York, NY, United States; ^3^Pathology Department, Tufts Cummings School of Veterinary Medicine, North Grafton, MA, United States; ^4^Nanostring Technologies, Seattle, WA, United States; ^5^Department of Clinical Sciences, Tufts Cummings School of Veterinary Medicine, North Grafton, MA, United States; ^6^Department of Microbiology, Immunology and Pathology, Colorado State University Veterinary Diagnostic Laboratory, Fort Collins, CO, United States; ^7^Department of Population and Quantitative Health Sciences, UMass Chan Medical School, Worcester, MA, United States

**Keywords:** cutaneous T cell lymphoma (CTCL), epitheliotropic lymphoma (EL), interface dermatitis (ID), dog (canine), cathepsin W (CTSW), T cell receptor associated transmembrane adaptor 1 (TRAT1), killer cell lectin like receptor K1 (KLRK1), lymphotactin/XCL1/XCL2

## Abstract

Cutaneous T-cell lymphoma (CTCL) is an uncommon type of lymphoma involving malignant skin-resident or skin-homing T cells. Canine epitheliotropic lymphoma (EL) is the most common form of CTCL in dogs, and it also spontaneously arises from T lymphocytes in the mucosa and skin. Clinically, it can be difficult to distinguish early-stage CTCLs apart from other forms of benign interface dermatitis (ID) in both dogs and people. Our objective was to identify novel biomarkers that can distinguish EL from other forms of ID, and perform comparative transcriptomics of human CTCL and canine EL. Here, we present a retrospective gene expression study that employed archival tissue from biorepositories. We analyzed a discovery cohort of 6 canines and a validation cohort of 8 canines with EL which occurred spontaneously in client-owned companion dogs. We performed comparative targeted transcriptomics studies using NanoString to assess 160 genes from lesional skin biopsies from the discovery cohort and 800 genes from the validation cohort to identify any significant differences that may reflect oncogenesis and immunopathogenesis. We further sought to determine if gene expression in EL and CTCL are conserved across humans and canines by comparing our data to previously published human datasets. Similar chemokine profiles were observed in dog EL and human CTCL, and analyses were performed to validate potential biomarkers and drivers of disease. In dogs, we found enrichment of T cell gene signatures, with upregulation of *IFNG*, *TNF*, *PRF1*, *IL15*, *CD244*, *CXCL10*, and *CCL5* in EL in dogs compared to healthy controls. Importantly, *CTSW*, *TRAT1* and *KLRK1* distinguished EL from all other forms of interface dermatitis we studied, providing much-needed biomarkers for the veterinary field. *XCL1*/*XCL2* were also highly specific of EL in our validation cohort. Future studies exploring the oncogenesis of spontaneous lymphomas in companion animals will expand our understanding of these disorders. Biomarkers may be useful for predicting disease prognosis and treatment responses. We plan to use our data to inform future development of targeted therapies, as well as for repurposing drugs for both veterinary and human medicine.

## Background

Cutaneous T cell lymphomas (CTCL) are a heterogenous group of non-Hodgkin’s lymphomas characterized by the proliferation of neoplastic T-lymphocytes in the skin (1). The most common subtype in humans is mycosis fungoides (MF), which is known for its progression of three stages: patches, plaques, and tumors of which their mushroom-like appearance inspires the name (2). CTCLs often present with patches, plaques, ulcerations, or other skin rashes, and can evolve into cutaneous tumors and/or progress to visceral involvement. In the United States, the overall annual age-adjusted incidence of CTCL was 6.4 per million persons over the time period of 1973 to 2002, with an annual incidence increase of 2.9 × 10^−6^ (3). The incidence is greater in males than in females, and in Black people than in White people (3).

Dogs also develop cutaneous lymphomas, including T and B cell lymphomas (4–6). In canine cutaneous epitheliotropic lymphoma (EL), neoplastic lymphocytes infiltrate the skin and mucosa (7). Canine epitheliotropic T cell lymphoma (T-EL) has different subtypes when described with the same standards as humans, including MF, Sézary syndrome, and pagetoid reticulosis (8). The disease progression in both human CTCL and canine T-EL are very similar, and usually involve progression from patch stage to plaque stage to tumor stage. Clinical presentations of both diseases involve exfoliative erythroderma, ulceration, depigmentation, plaques, and nodules, and both diseases are difficult to diagnose in earlier stages due to similar clinical presentation to inflammatory or benign processes (6). While CD4^+^ helper T-cells predominantly drive disease in human CTCL, canine T-EL is predominantly a disease of CD8^+^ cytotoxic T-cells (8).

One challenge in the veterinary field that remains is distinguishing early-stage EL in dogs from other immune-mediated interface dermatitis conditions (6). Both clinical and histopathological features of early-stage EL can be mistaken for atopy or other immune-mediated processes (7, 9). A reliable biomarker to differentiate EL from other interface diseases would enable initiation of treatment at earlier stages of disease. A recently published study examined transcriptional differences between canine EL and immune-mediated dermatoses using RNA sequencing (10). Here, we employ microarray technology and demonstrate that *CTSW*, *TRAT1*, *KLRK1* and *XCL1*/*2* probes may be used to distinguish EL from other forms of interface dermatitis in dogs. Further, comparative immunology approaches to assess the gene expression patterns of canine EL to human CTCL revealed shared signatures, indicating that these may also serve as biomarkers of some forms of human CTCL. This may be particularly important to distinguish CTCL from clinical mimickers, thereby preventing misdiagnosis in both veterinary and human patients.

## Methods

### Study design

The goals of this study were to: (1) define the transcriptome of EL using RNA isolated from diagnostic archival tissue biopsies using NanoString and (2) determine whether gene biomarkers can be used to distinguish EL from ID.

### Clinical samples

Skin biopsies from the biorepository at Tufts Cummings School were selected based on pathology reports. H&E sections were reexamined by a board-certified veterinary pathologist to confirm diagnoses and absence of obvious infectious disease, and clinical notes were reexamined by a board-certified veterinary dermatologist. Healthy control skin samples were obtained from leg margin biopsies from amputations. For the discovery cohort, six epitheliotropic lymphoma samples were obtained from shave and/or punch biopsies of dogs as noted in the case presentation section. Samples were obtained as part of routine medical care under the guidance of a veterinarian at the Foster Hospital for Small Animals at Cummings School of Veterinary Medicine, spanning the years 2011–2019. For the validation cohort, eight EL samples were obtained from the Colorado State University Veterinary Diagnostic Laboratory, seven of which yielded enough RNA for downstream analyses. Cases were reviewed by a board-certified veterinary pathologist to confirm diagnosis.

### Isolation of RNA from FFPE blocks

Thirty μm curls were cut from FFPE blocks and stored in Eppendorf tubes at ambient temperature. RNA was isolated using the Qiagen FFPE RNeasy kit per the manufacturer directions. Briefly, razor blades were treated with RNase, excess paraffin was removed, and tissues were sliced into thin strips (5 μm) to create more surface area prior to incubation with deparaffinization solution (Qiagen). The manufacturer protocol was followed and RNA was quantified using a Nanodrop spectrophotometer (Fisher Scientific).

### Nanostring cartridge and processing

A custom designed Nanostring canine gene panel of 160 genes including cytokine, chemokine, and immune genes, as well as skin and immune cell specific transcripts was created as previously described (11). We used *B2M*, *RPL13A*, *CCZ1* and *HPRT* as housekeeping genes for this study. For the validation cohort, the NanoString canine immune-oncology (IO) panel was used. RNA (150 ng/assay) was hybridized for 18 h using a BioRad C1000 touch thermal cycler, and samples were loaded into Nanostring cartridges and analyzed with a Sprint machine according to manufacturer’s instructions. Gene expression data are deposited on GEO under Accession # GSE213087.

### nSolver analysis

NanoString’s software, nSolver was used for all NanoString analysis. Raw counts were plotted with GraphPad Prism. Advanced analysis was used for the “cell type score,” which is a summary statistic of the expression of the marker genes for each cell type. It is the geometric mean of the log2-transformed normalized counts for each set of marker genes. NanoString validated these cell Type Scores against FACS and IHC (12).

### IHC

IHC was performed on 5 μm sections using rabbit-anti-human/mouse/rat CD244 (catalog #521141; US Biological), rabbit-anti-canine/human CTSK/CTSO/CTSX/CTSO2 (catalog # 139662; US Biological) or isotype control (Biolegend catalog # 910801) at 1:100 dilution using a Dako automated slide staining machine. Briefly, antigen retrieval was performed using Retrievagen A (pH 6.0; BD Pharmingen) in the microwave set to high setting twice for 10 min. Primary antibodies were incubated at room temperature for 60 min. The DAKO Duel link system (code K4065) was used for secondary antibody staining for 45 min at room temperature per the manufacturer’s protocol. All sections were counterstained with hematoxylin. H&E images were taken using an Olympus BX51 microscope with Nikon NIS Elements software version 3.10, and IHC images were taken using an Olympus BX40 microscope with cellSens Entry software version 1.14.

### qPCR

cDNA synthesis was performed on RNA extracted from FFPE or frozen lymphoma samples using BioRad iScript cDNA synthesis kit per manufacturer instructions. qPCR for *CTSW* or *GAPDH* housekeeping gene was performed with technical duplicates on 3 biologic samples per condition using iTaq universal SYBR green supermix per the manufacturer’s protocols on either a QuantStudio (Applied Biosystems) or BioRad CFX96 machine. Relative copy numbers were calculated using the formula in Excel “=(10^((Ct value *_CTSW_* – 40)/−3.32)))/(10^((Ct value _Average *GAPDH*_ – 40)/−3.32))).”

### Comparison to human CTCL

The human CTCL dataset from Nielsen et al. (13) (GSE143382; https://www.ncbi.nlm.nih.gov/geo/query/acc.cgi?acc=GSE143382) was analyzed using Geo2R. Gene lists were truncated in Microsoft Excel using the formula “=IF(ISERROR(VLOOKUP(cell,reference,1,FALSE)),FALSE,TRUE)” and shared DEGs between human and canine were analyzed with BioVenn (14).

### Statistics

Differentially expressed genes (DEGs) were analyzed using Rosalind software and/or nSolver software. We also analyzed raw and/or normalized counts between groups using nSolver and GraphPad Prism software version 9 to examine potential differences in previously identified genes pertinent to EL and CTCL pathogenesis. Normality tests were performed in GraphPad Prism. Normally distributed data were analyzed using student’s *t*-test and non-normally distributed data were analyzed using Mann–Whitney *U* test. Receiver operator characteristic (ROC) curves were calculated in GraphPad Prism. Multi-ROC curves were calculated by a biostatistician in SPSS. A statistically significant difference was considered as *p* < 0.05.

## Results

### Canine EL exhibits differentially expressed immune and skin genes compared to healthy controls

**Figure 1:**
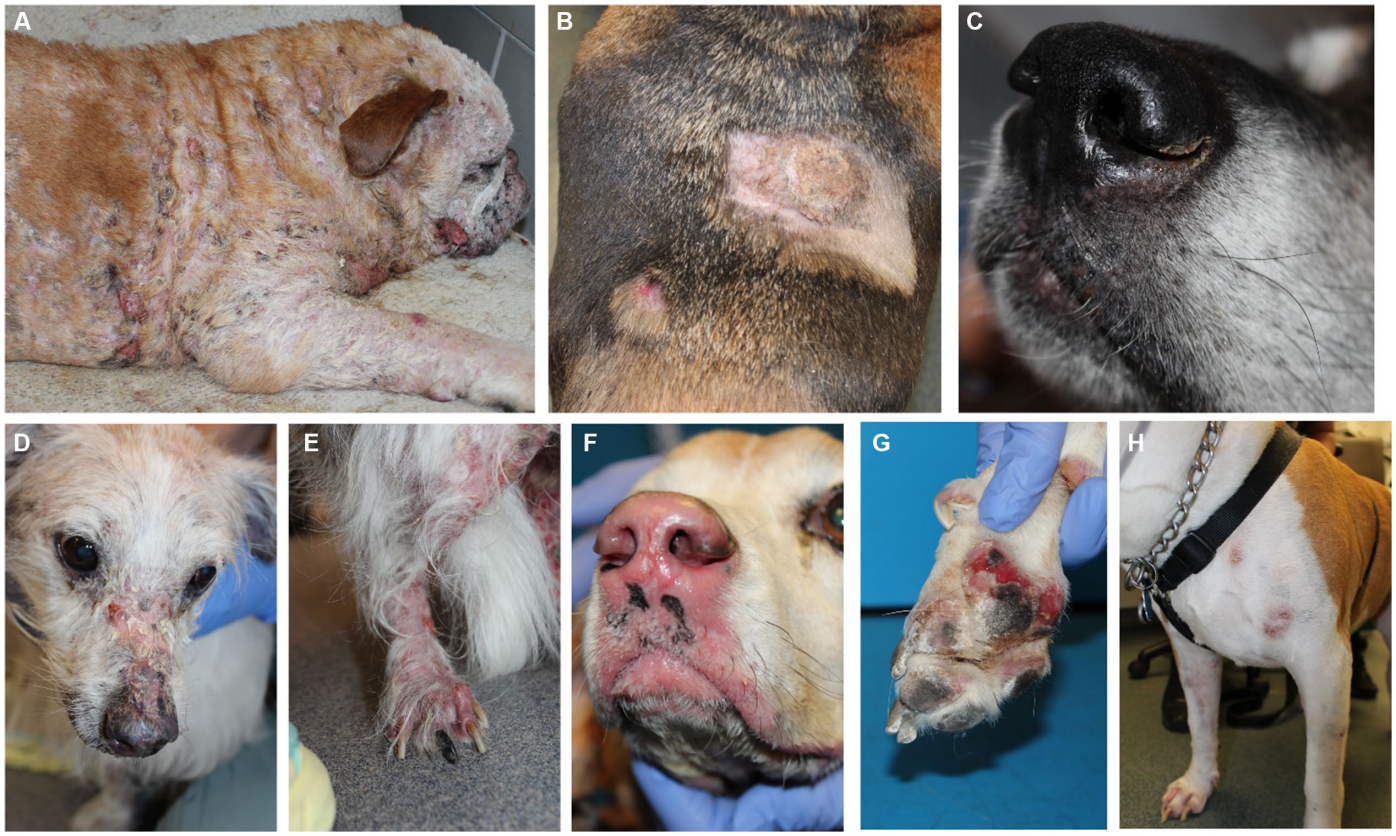
Clinical presentations of epitheliotropic lymphoma in dogs. **(A)** Widely disseminated lesions with hair loss associated with severe scale-crusting. **(B)** Erythematous alopecia patch and hyperkeratotic plaque lesions on the dorsum. **(C)** Mild crusting with depigmentation, loss of nose cobblestone appearance and erythema on the muzzle. (**D)** Alopecia, erythema and crusting on the nasal planum and **(E)** hind legs. **(F)** Depigmentation, loss of nose cobblestone appearance and erythema on the muzzle, which can be mistaken for other immune-mediated processes. **(G)** Footpad involvement with crusting and ulcerations. **(H)** Multifocal lesions with erythema and crusting and without notable hair loss. Please note that breed is not associated with a particular presentation of the disease.

**Figure 2:**
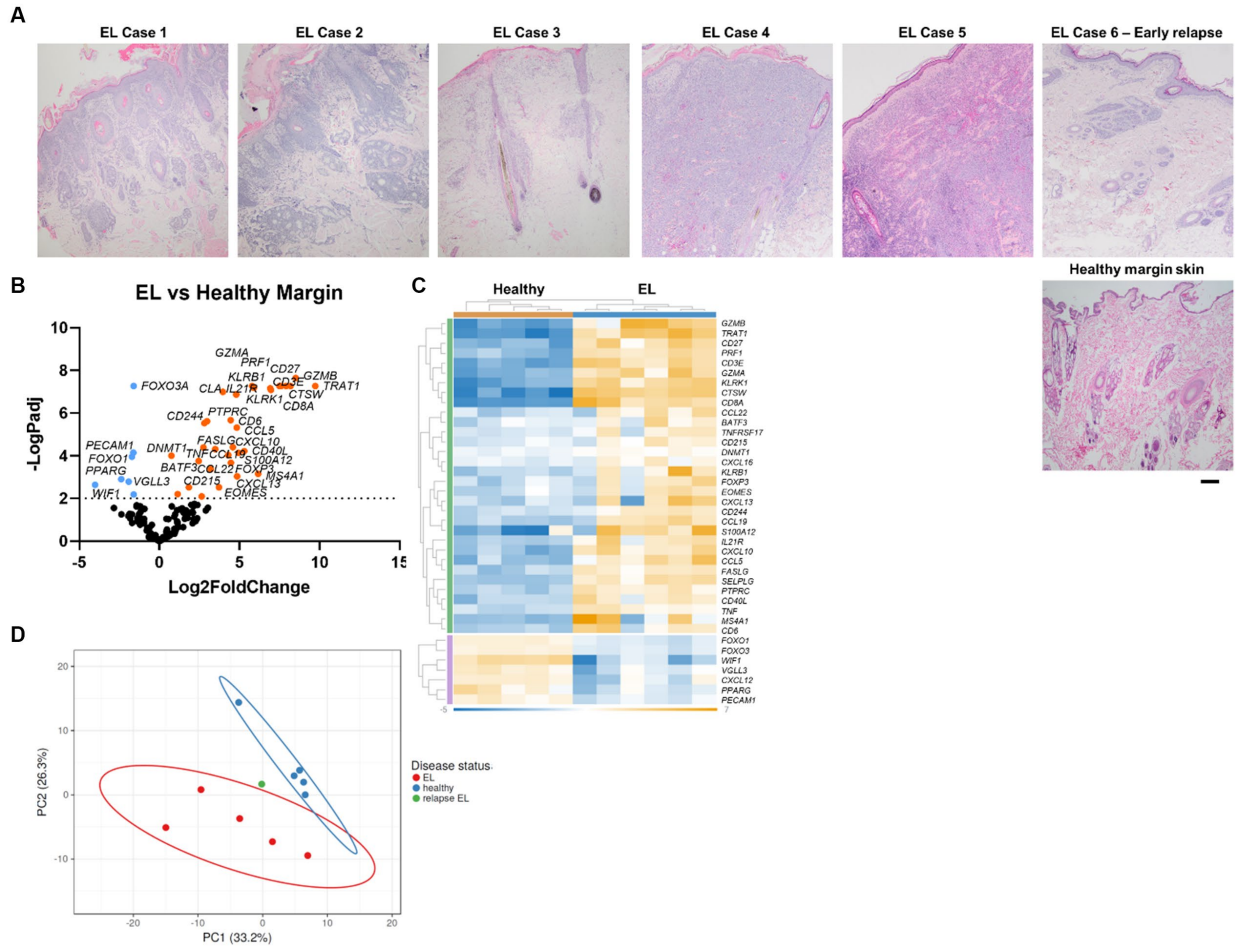
Gene expression in EL versus healthy margin controls reveals CD8^+^ cytotoxic T cell signatures. **(A)** Sample H&E photomicrographs of healthy and EL tissue (scale bar 200 μm). **(B)** Volcano plot of differentially expressed genes (DEGs) between healthy and EL. **(C)** Heatmap of DEGs generated with Rosalind software. **(D)** Principal component analysis (PCA) of cases versus healthy margins generated with ClusVis software (*n* = 6 EL and 5 healthy controls, *p*_adj_ <0.05 considered significant).

Canine EL can present with different features including depigmentation, crusting, erythema, ulceration and/or alopecia (Figure 1). We analyzed residual tissue blocks from diagnostic biopsies to assess gene expression (Figure 2A; Table 1). Using the NanoString nCounter platform, which is optimized for FFPE RNA analysis, we performed targeted transcriptomics studies on 160 custom curated genes from lesional skin biopsies from 6 canine EL cases and 5 healthy canine cases. Our NanoString probeset targets included cytokine, chemokine, and immune related genes, as well as skin associated genes and neuroendocrine genes. Advanced cell type analysis revealed cytotoxic T cells were significantly abundant (*p* = 0.0001; not shown). Comparing the EL samples to healthy margins revealed 32 upregulated DEGs and 7 down regulated differentially expressed genes (DEGs) with *p*_adj_ <0.01 (Figures 2B,C). An array of genes involved in cytotoxic processes were all upregulated, including *GZMA*, *KLRB1*, *KLRD1*, *KLRK1*, and *PRF1*. *CPA3*, which is involved in proteolysis and degradation of endogenous proteins, was upregulated in EL lesions. Principal component analysis revealed that EL cases could be readily distinguished from healthy controls in 95% confidence intervals, with the exception of one early relapse case which fell in between the EL and healthy skin gene signatures (Figure 2D).

**Table 1:** Discovery cohort signalments.

Case and diagnosis	Signalment^a^	Breed	Relevant clinical and histopathological findings
EL case 1	11 yo, MN	Dachshund	Patch/plaque stage
EL case 2	13 yo, MN	Labrador Cross	Multifocal ulceration with moderate neutrophilic inflammation
EL case 3	11 yo, FS	Labrador Retriever	Significant eosinophilic infiltrate accompanying the neoplastic population; multifocal ulceration, serocellular crusts and intracorneal neutrophilic pustules
EL case 4	9 yo, FS	Bloodhound	Significant dermal neoplastic involvement
EL case 5	11 yo, FS	Olde English Bulldogge	Significant dermal neoplastic involvement with epitheliotropism restricted to the adnexa with relative sparing of the epidermis
EL case 6	13 yo, MN	Golden Retriever	Early stage epitheliotropic lymphoma with minimal to absent dermal involvement. This is a recurrence from epitheliotropic lymphoma that had undergone remission
Healthy 1	8 yo, FS	Labrador Retriever	NA
Healthy 2	11 yo, FS	Siberian Husky Cross	NA
Healthy 3	11 yo, MN	Golden Retriever	NA
Healthy 4	12 yo, MN	German Shepherd Cross	NA
Healthy 5	6 yo, FS	Alaskan Malamute	NA

FS, female spayed; MN, male neutered.

a Age at time of biopsy.

### Gene biomarkers can distinguish EL from other interface dermatitis conditions

**Figure 3:**
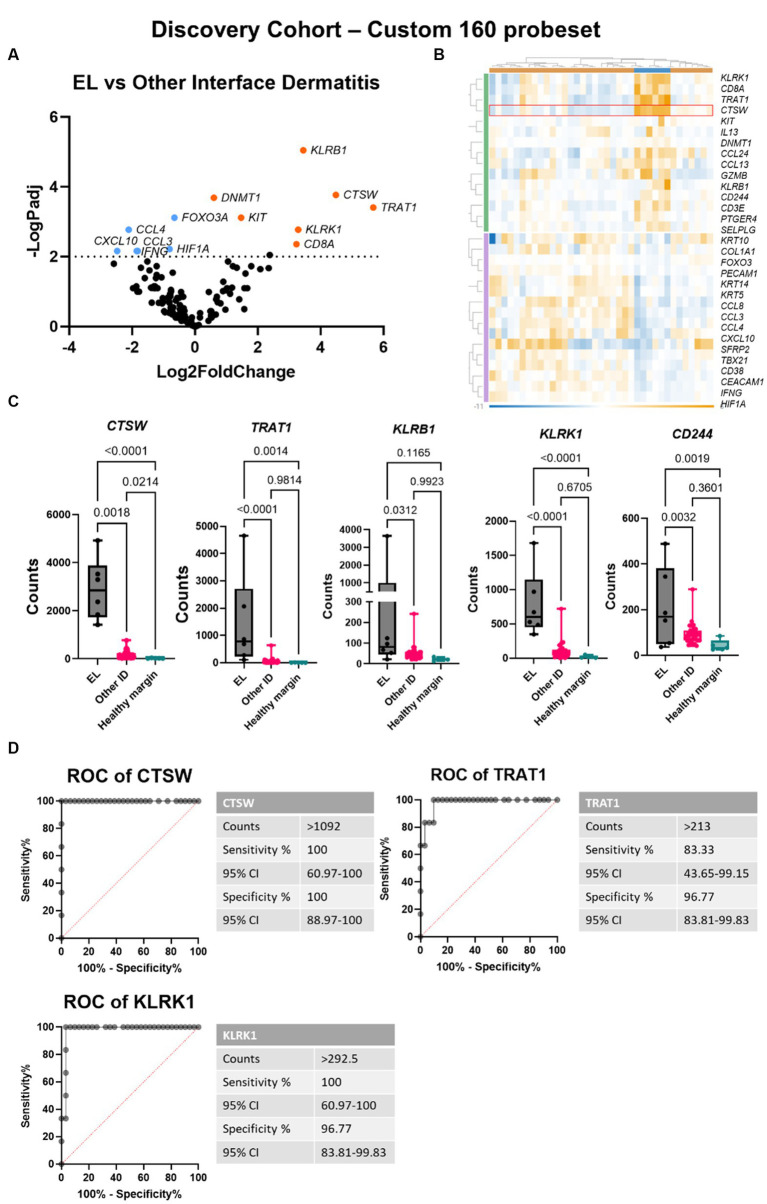
Gene expression analysis reveals *CTSW*, *TRAT1* and *KLRK1* distinguish between epitheliotropic lymphoma and interface dermatitis. **(A)** Volcano plot of EL versus other interface dermatitis conditions including cutaneous lupus erythematosus, pemphigus, and erythema multiforme spectrum disorders. **(B)** Heatmap generated in Rosalind software. **(C)** Raw counts of the top DEGs between EL and ID (one-way ANOVA with Tukey’s post tests significant as indicated). **(D)** Receiver operator characteristic (ROC) curves of *CTSW*, *TRAT1* and *KLRK1* (*n* = 6 EL and 31 other interface dermatitis conditions; *p*_adj_ <0.05 considered significant).

Next, we compared EL gene expression to other forms of interface dermatitis (ID) including lupus erythematosus, pemphigus subtypes, and erythema multiforme spectrum conditions. Seventeen genes were significantly downregulated and 14 were significantly upregulated with *p*_adj_ <0.05 (Figures 3A,B). To evaluate whether any of these DEGs could serve as potential diagnostic biomarkers for EL, we analyzed RNA counts of the highest DEGs singly (Figure 3C). Of these, *CTSW* exhibited no overlap between cases and cleanly distinguished EL from other potential clinical mimickers. Receiver operator characteristic (ROC) curves of *CTSW* was 100% sensitive and specific for counts >1,092 (Figure 3D). *TRAT1* and *KLRK1* were also highly significant (*p* < 0.0001), and ROC analysis revealed 83.3% sensitivity and 96.77% specificity for *TRAT1* counts >213, and 100% sensitivity and 96.77% specificity for *KLRK1* counts >292.5.

**Figure 4:**
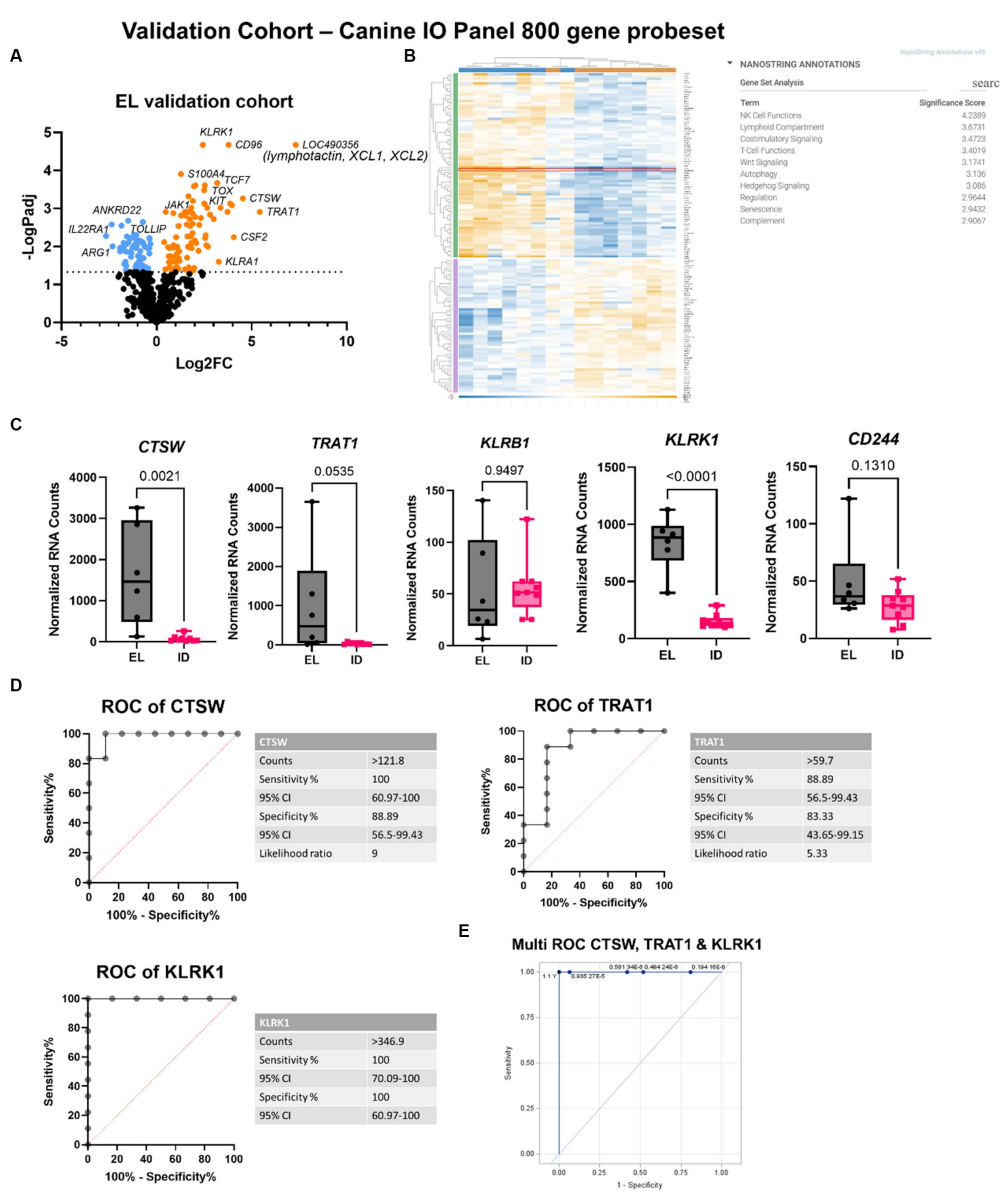
Validation cohort confirms *CTSW*, *TRAT1*, *KLRK1* and identifies *XCL1*/*XCL2* as potential biomarkers for EL. **(A)** Volcano plot of EL versus other interface dermatitis conditions. **(B)** Heatmap and gene set analysis generated in Rosalind software. **(C)** Raw counts of the top DEGs between EL and ID (one-way ANOVA with Tukey’s post tests significant as indicated). **(D)** Receiver operator characteristic (ROC) curves of *CTSW* and *TRAT1*. **(E)** Multi-ROC curve combining *CTSW*, *TRAT1* and *KLRK1* normalized counts for distinguishing EL from ID in the discovery and validation cohorts (discovery cohort: *n* = 6 EL and 31 other interface dermatitis conditions; validation cohort: *n* = 6 EL and 9 other interface dermatitis conditions; *p*_adj_ <0.05 considered significant).

To confirm this finding, we performed analysis on a validation cohort of 6 EL and 9 interface dermatitis (ID) samples using the NanoString canine IO panel (Figures 4A,B; Supplementary Table S1). *CTSW* exhibited 100% sensitivity and 88.9% specificity to distinguish the two conditions at a count >121.8 (Figures 4C,D). *TRAT1* exhibited 88.89% sensitivity and 83.33% specificity to distinguish EL from ID at a count >59.7. *KLRK1* was 100% sensitive and specific at counts >346.9. The difference in absolute counts between the discovery cohort and the validation cohort is likely due to the 2 different gene panels used (160 custom gene codeset versus canine IO ~800 gene codeset). We noted that an additional biomarker was identified by the canine IO panel: lymphotactin also called *XCL1*/*XCL2*, though we were unable to verify this gene in our discovery cohort because it was not included in the 160 gene codeset. Last, we examined whether combining biomarkers could more accurately identify EL compared to each gene alone. Multi-biomarker ROC analysis revealed that combining *CTSW*, *TRAT1* and *KLRK1* is highly sensitive and specific for identifying EL as compared to other forms of ID (Figure 4E).

### Comparative analysis of canine EL and human CTCL reveals shared inflammatory and immunoregulatory gene expression signatures

**Figure 5:**
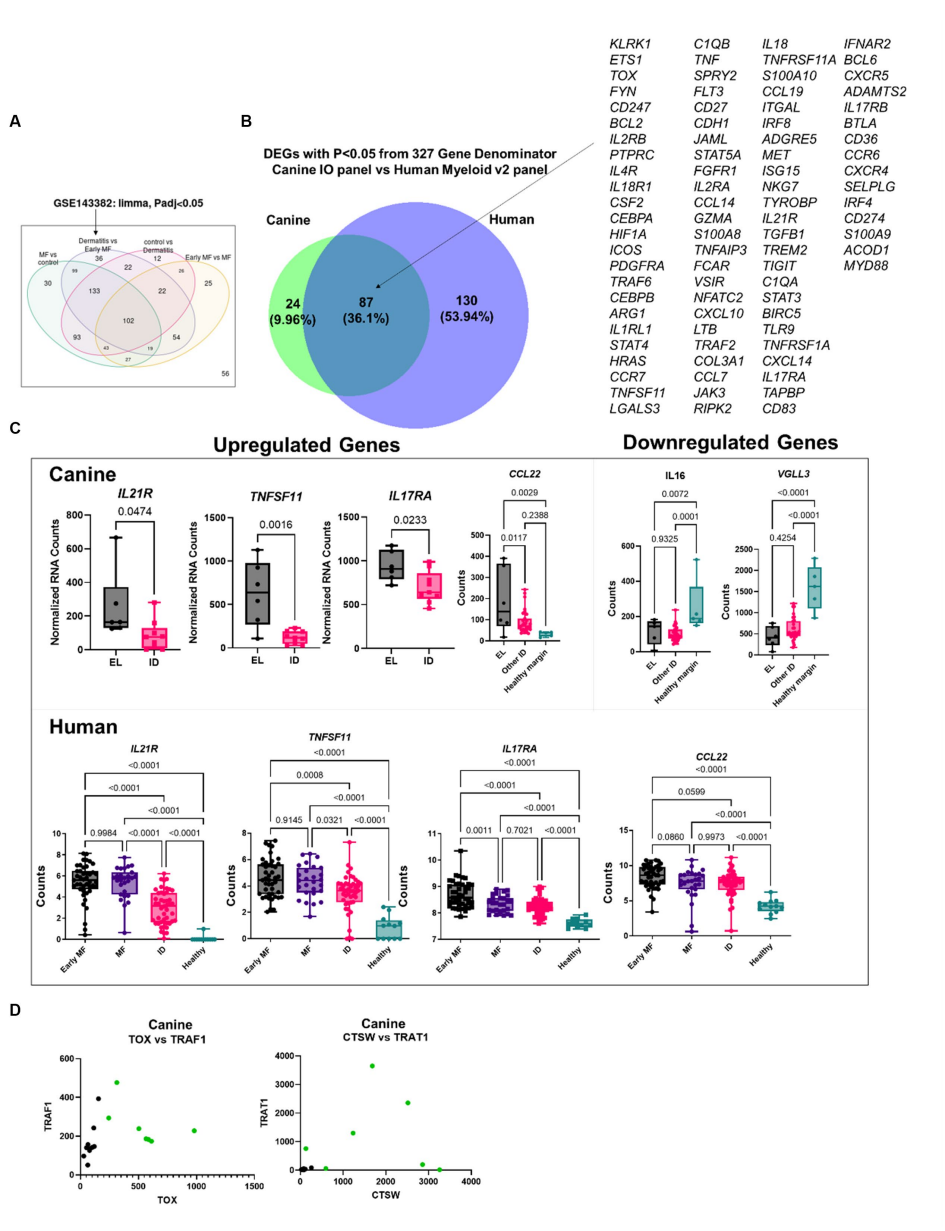
Comparative transcriptomics of human MF and canine EL versus ID DEGs. **(A)** Geo2R reanalysis of GSE143382 human MF dataset. DEGs from dermatitis vs. early MF were used as they represent the closest clinical match. **(B)** DEG overlap between the canine validation cohort (canine IO panel) and human dataset (myeloid v2 panel) using the 327 common denominator genes from the respective NanoString probesets. **(C)** Comparison of previously published up-and downregulated genes in canine and human datasets. **(D)** Analysis of *TOX* vs. *TRAF1*, which was used to distinguish human MF from ID, or *CTSW* vs. *TRAT1* in canine samples.

We also compared our EL findings to a previously published human CTCL dataset [Human Dataset GSE143382 (13)]. We focused on the DEGs between early MF and ID (Figure 5A). First, we truncated the datasets to a common denominator gene list of 327 based on the NanoString panels (canine IO and human Myeloid v2). Next, we compared which of these genes were significantly differentially expressed (*p* < 0.05), identifying 87 overlapping DEGs (Figure 5B). We examined specific genes identified in our DEG overlap as well as other published genes of interest and found similar expression trends in human and canine datasets (Figure 5C). We examined whether the two-gene classifier identified in human MF by Nielsen et al. (13) would also distinguish canine EL from ID, and found that while *TOX* and *TRAF1* could separate the cases (green) from controls (black), better separation is achieved with *CTSW* and *TRAT1* (Figure 5D).

### Cathepsins and CD244 are expressed at the protein level in canine EL and ID lesions, but RNA probe is superior to antibody for distinguishing the conditions

To confirm protein level expression of key genes, we performed IHC for CTSK/CTSO/CTSX/CTSO2 cathepsin family members using an antibody reactive to dog/human, and CD244 using antibodies that react to human/mouse/rat proteins with predicted homology to canine amino acid sequences, as no canine specific antibodies for CTSW or CD244 are commercially available. IHC staining did not differentiate EL from ID lesions, indicating possible cross-reactivity of the antibodies to other epitopes and/or discordance between RNA transcripts and protein level expression in the different conditions (Supplementary Figures S1, S2). Similarly, qPCR for *CTSW* was not effective at distinguishing EL from ID in FFPE samples, which may be due to the highly fragmented RNA (Supplementary Figure S3; Supplementary Table S2). To this end, we developed a truncated microarray diagnostic tool with our 4 biomarkers and 5 housekeeping genes, which was able to sensitively and specifically distinguish EL from ID in FFPE samples (Supplementary Figure S4).

## Discussion

Here, we identified biomarkers that can sensitively and specifically detect EL to distinguish it from other forms of ID. These biomarkers may have biological relevance to tumor biology, as we will delineate below.

*CTSW* is a member of the cathepsin S family that is a papain-like protease. Cathepsins have been reported to regulate cancer progression and therapeutic responses (15). Cathepsin S is upregulated in follicular lymphoma, and an activating mutation Y132D drives lymphomagenesis through alterations in antigen presentation (16) and a pro-tumorigenic microenvironment (17). Cathepsin W is known to be expressed in human CD8^+^ T cells (18) and natural killer (NK) cells (19, 20). Similarly, family member cathepsin G is 2-fold upregulated in early disease stages (IA/IB) (21). In breast cancer, increased expression of cathepsin B and cathepsin L is associated with poorer prognosis, greater mortality, and greater disease metastases (15). In colorectal cancer, elevated cathepsin B and cathepsin L are associated with increased disease metastasis and poorer prognosis (22), while elevated cathepsin S predicts both decreased survival when treated with surgery alone, with potential benefit from adjuvant 5-fluorouracil and folinic acid treatment. High cathepsin B expression in lung, ovarian, pancreatic neuroendocrine cancers and pancreatic adenocarcinomas, is negatively correlated with survival and positively associated with recurrence, invasion, and/or tumor grade. Upregulation of cathepsin K in osteosarcomas predicts poor prognosis and disease metastasis. Generally, it seems that the tumor microenvironment helps activate cathepsins, which in turn activate oncogenesis: mutant HRAS in mammary epithelial cells upregulates *CTSB* and *CTSL*; the *HER2* oncogene drives expression of *CTSB* through the transcription factor myeloid zinc finger 1 (MZF1), and *CTSB* is a functional driver of the invasive phenotype.

*TRAT1* is a 30 Kd type III transmembrane protein expressed by human T lymphocytes and natural killer (NK) cells. *TRAT1* regulates T-cell receptor expression (23). It consists of an extracellular domain, transmembrane region, and cytosolic tail. TRAT1 facilitates CTLA-4 shuttling from the trans Golgi network to the T-cell surface where it stabilizes the T-cell antigen receptor and CD3 complex. TRAT1 knockdown experiments demonstrated reduced CTLA-4 mediated cytokine release as well as CTLA-4 cell surface expression and subcellular distribution. When TRAT1 is overexpressed in Jurkat T cells, there is an increase in T-cell receptor expression, however this is not the case in regular T cells (24). By contributing to the structural integrity of the TCR/CD3 complex, TRAT1 has a significant role in TCR functions, which include triggering antigen-specific T-cell responses (25). In non-blood cancers (solid tumors), *TRAT1* expression is important for a good prognosis. However, in T cell lymphomas, *TRAT1* can be highly expressed in tumor cells. It remains unknown how this relates to prognosis.

*KLRK1* is also known as NKG2D. It is a killer type lectin receptor expressed on NK cells and cytotoxic T cells (26). *KLRK1* is upregulated in human peripheral T cell lymphomas (27). A recent study demonstrated that benign T cells drive inflammation in MF tumors in humans (28): an influx of CD8^+^ T cells following immunotherapy in CD4-driven tumors was protective. Therefore, caution should be exercised in fully characterizing the tumor (e.g., CD4 or CD8; does the tumor itself bear *KLRK1,* or is it expressed by the infiltrating T cells (29)) to ascertain whether *KLRK1* both as a biomarker and a potential treatment target is beneficial or detrimental (28).

*LOC490356* was a top DEG in our validation cohort. This locus encodes *lymphotactin*, also known as *XCL1*/*XCL2*. This chemokine is homeostatically expressed by NK cells and has antimicrobial activity (30). *XCL1* is downregulated in human Sezary syndrome (31, 32), but was expressed by T-3B cells in a case report of a patient with concurrent T and B cell cutaneous lymphomas (33) and by T cells in a patient with lymphoproliferative disorder (34). As with *KLRK1*, it is unclear whether *XCL1* could be protective or pathogenic in the EL setting. In murine tumor models, injection of XCL1-expressing myeloma cells in Balb/c and nude mice resulted in tumor regression (35). An XCL1 fusion peptide improved tumor rejection in a mouse B16 model of melanoma via recruitment of XCR1^+^ dendritic cells to the tumor (36). This also raises the point that some of the associated biomarkers identified for EL and other forms of CTCL may be expressed by infiltrating immune cells and not the tumor itself. Nevertheless, these seem to serve as sensitive and specific biomarkers for identifying early stage EL apart from other forms of interface dermatitis.

Other biologically relevant genes identified in our datasets correspond to both tumor and immune cell function. *CD6*, *CD86*, and *CD8A* were all upregulated, and are implicated in T-cell activation and regulation. *MS4A1*, a gene that encodes the B-cell marker CD20, was upregulated in EL. *PTPRC* was also upregulated, and is essential for T- and B-cell antigen receptor signaling. Immunoregulatory cytokines and their receptors were upregulated including *IL6* and *IL21R*. *S100A12* regulates inflammatory and immune responses, and was upregulated in EL. Tumor necrosis factor alpha (TNF-alpha) has been implicated in the pathogenesis of CTCL (37). *TNF*, *TNFRSF17*, *CD27*, and *FASLG* were all significantly upregulated in canines with EL. *CD27*, a TNF family member required for the maintenance of T-cell immunity, was significantly upregulated. *FoxP3* has been reported to be variably expressed depending on the stage of disease in CTCL (38). *FoxP3* was not significantly differentiated in the EL canines compared to the healthy controls; however two canines, Sample 3 and Sample 4, had relatively greater counts than all other cases. They represent cases with significant dermal neoplastic involvement and patch/plaque stage, respectively. Last, given that *CCL22* was upregulated, *CCR4* depleting antibodies like mogamulizumab may be helpful for treating canine EL.

We also observed downregulated genes that may be biologically significant in EL. *VGLL3* has been recognized as a tumor suppressor gene in serous ovarian carcinomas (39, 40) and stomach adenocarcinoma (41). *VGLL3*, which regulates the Hippo pathway (42), was downregulated in EL skin. *IL16* was also downregulated, reflecting the pattern observed in severe Sézary syndrome (43). Reintroduction of pro-IL16 in MOLT4 tumors can induce regression in nude mice (44). Therefore, adding back tumor suppressor genes may serve as a therapeutic option for EL and should be considered in future veterinary clinical trials.

Forms of CTCL that express CD8^+^ phenotype in humans have been reported (38): in some rare cases, well-defined types of CTCL (such as MF) express CD8^+^ and have similar clinical presentation and disease prognosis as the more common CD4^+^ cases (45). Outside of these, studies suggest separate groups of more aggressive CD8^+^ cytotoxic CTCLs (46), including an epidermotropic type and a panniculitis-like subcutaneous T cell lymphoma type. Therefore, it is possible that, genetically and/or transcriptionally, canine EL matches human CD8^+^ lymphomas more closely than MF as a general subtype. Future comparative oncology studies should further characterize these rare CD8^+^ tumors to better understand which condition is most closely modeled in dogs.

In conclusion, comparative studies investigating the conservation of oncogenic processes and tumor immune landscapes across species are important for identifying biomarkers and treatment targets. Further investigation is warranted to expand our understanding of these disorders and predict disease prognosis and treatment responses. We plan to use our newly identified biomarkers to diagnose and track dogs with EL in veterinary clinical trials, and our long-term goal is to develop targeted therapies for both veterinary and human CTCLs.

## Data availability statement

The datasets presented in this study can be found in online repositories. The names of the repository/repositories and accession number(s) can be found at: https://www.ncbi.nlm.nih.gov/geo/, GSE213087.

## Ethics statement

Ethical approval was not required for the study involving humans in accordance with the local legislation and institutional requirements. Written informed consent to participate in this study was not required from the participants or the participants’ legal guardians/next of kin in accordance with the national legislation and the institutional requirements. The animal studies were approved by Tufts Cummings School of Veterinary Medicine IACUC. The studies were conducted in accordance with the local legislation and institutional requirements. Written informed consent was obtained from the owners for the participation of their animals in this study.

## Author contributions

JR: conceptualization and project administration. NR and JR: methodology. CD: software. CP-M, AR, AN, HG, KH, BB, and JR: validation. JO, DM, NW, BB, and JR: formal analysis. C-PM, NR, JR, CD, and LW: investigation. NR, KH, CL, and JR: resources. NR, RA, JO, CP-M, KH, and JR: data curation. JO, AN, and JR: writing—original draft. JO, AN, DM, NW, AR, CP-M, LW, CD, HG, NR, KH, BB, CL, RA, and JR: writing—review & editing. JO, CP-M, NW, DM, and JR: visualization. JR, RA, and NR: supervision. JR and JO: funding acquisition. All authors contributed to the article and approved the submitted version.

## Abbreviations

CTCL: Cutaneous T cell lymphoma
DEGs: Differentially expressed genes
EL: Epitheliotropic lymphoma
FFPE: Formalin fixed paraffin embedded
ID: Interface dermatitis
MF: Mycosis fungoides
