# Supplementary material for: Cathepsin W, T-cell receptor-associated transmembrane adapter 1, lymphotactin and killer cell lectin like receptor K1 are sensitive and specific RNA biomarkers of canine epitheliotropic lymphoma
Source: Front Vet Sci. 2023 Nov 3;10:1225764. doi: 10.3389/fvets.2023.1225764 (PMC10654980; doi:10.3389/fvets.2023.1225764)
Supplement: Supplementary file 1 [file Data_Sheet_1.docx]

**Table S1. Validation cohort signalments.**

| **Case & Diagnosis** | **Signalment*** | **Breed** |
| --- | --- | --- |
| EL Validation Case 1 | 13yo spayed Female | Golden Retriever |
| EL Validation Case 2 | 17yo spayed Female | Yorkshire Terrier |
| EL Validation Case 3 | 10yo spayed Female | Shih Tzu |
| EL Validation Case 4 | 10yo spayed Female | Labrador |
| EL Validation Case 5 | 8yo spayed Female | Australian Shepherd Cross |
| EL Validation Case 6 | 13.6yo neutered Male | Golden Retriever |

*age at time of biopsy

**Table S2. qPCR Primers used for these studies.**

| CTSW set 1 F | GCT TCC TGC TAC CCA CAC TT |
| --- | --- |
| CTSW set 1 R | AAG ATG TCC AGA CGA CGA GC |
| CTSW set 2 F | ACC CAT TCT TGG GGA ACA CC |
| CTSW set 2 R | AAC CCA CAA GCA GGA CAG AG |
| GAPDH F | GAT GGG CGT GAA CCA TGA G |
| GAPDH R | TCA TGA GGC CCT CCA CGA T |
